# Supplementary material for: Joint control of visually guided actions involves concordant increases in behavioural and neural coupling
Source: Commun Biol. 2021 Jun 29;4:816. doi: 10.1038/s42003-021-02319-3 (PMC8242020; doi:10.1038/s42003-021-02319-3)
Supplement: Supplementary file 2 — Description of Additional Supplementary Files [file 42003_2021_2319_MOESM2_ESM.pdf]

Description of Additional Supplementary Files

File Name: Supplementary Movie 1
Description: Example joint and solo control trials for a representative participant pair. For this participant pair, the green cursor and control cue indicated joint trials, and the red cursor and control cue indicated solo trials. “P1 View” shows a portion of Participant 1’s display. “P2 View” shows a portion of Participant 2’s display. Refer to Fig. 1 for a depiction of the display setup for the two participants in each pair. Playback speed corresponds to real-time speed.

File Name: Supplementary Movie 2
Description: Relative timing of cursor and eye-gaze movements. The video depicts all trials of a representative participant pair, with filled squares representing cursor positions and annuli representing eye-gaze positions. For solo trials, both participants’ visible cursors are plotted. For joint trials, only the visible joint cursor is plotted. The cursor data were resampled from 144 Hz to 120 Hz to match the sampling rate of the eye data. Playback speed is 25% of real-time speed. The time frame corresponds to the action epoch (0.0–2.5 s relative to the action cue to move).

File Name: Supplementary Movie 3
Description: Calculation of inter-cursor distance (ICD) on solo and joint control trials for a representative participant pair. “P1” indicates Participant 1’s cursor and “P2” indicates Participant 2’s cursor. “Joint” indicates the joint cursor. For illustrative purposes, white annuli indicate possible target positions. Note, however, that in actual trials only one target and one distractor (positioned 180º opposite) were displayed. ICD is shown here in pixel units. Note that the joint cursor only moved if both participants exerted sufficient force on their own video game controller (i.e., neither participant could be a “passenger” in joint control trials). Both visible and invisible cursors are depicted here, but in actual trials participants saw only their own cursor (P1 or P2) in solo trials (with joint invisible), and only the joint cursor in joint control trials (with P1/P2 cursors invisible). Playback speed corresponds to real-time speed.

File Name: Supplementary Data 1
Description: Data files for each of the main figure panels, analysis code showing how to plot the data , and plots corresponding to the analysis code.
