# Supplementary figures and images for: Joint control of visually guided actions involves concordant increases in behavioural and neural coupling
Source: Commun Biol. 2021 Jun 29;4:816. doi: 10.1038/s42003-021-02319-3 (PMC8242020; doi:10.1038/s42003-021-02319-3)

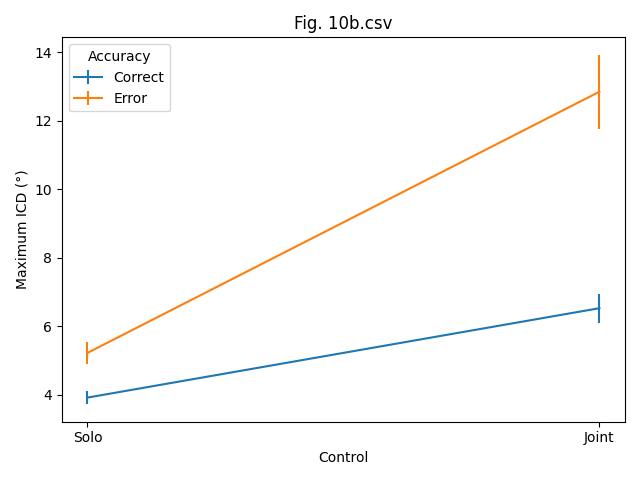

Supplement: Supplementary file 6 — Supplementary Data 1 [file 42003_2021_2319_MOESM6_ESM.zip › supplementary_data_publication/Fig. 10b.csv.png]

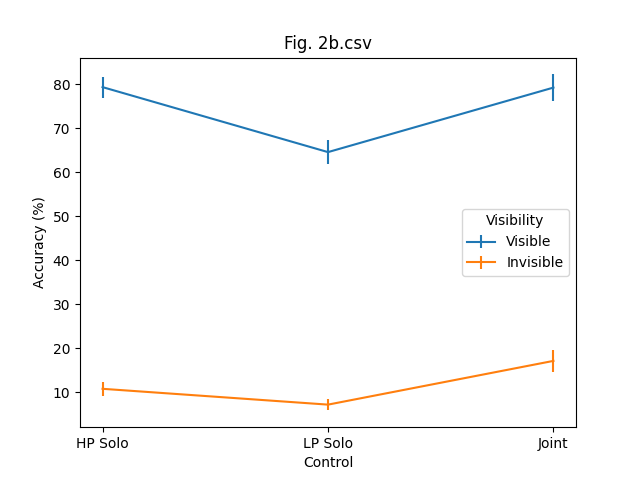

Supplement: Supplementary file 6 — Supplementary Data 1 [file 42003_2021_2319_MOESM6_ESM.zip › supplementary_data_publication/Fig. 2b.csv.png]

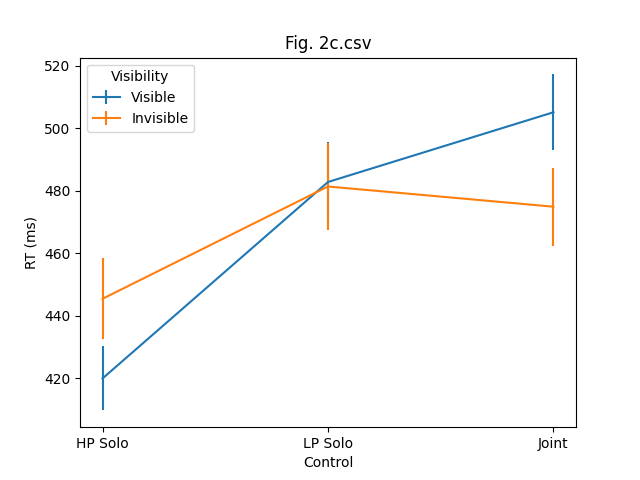

Supplement: Supplementary file 6 — Supplementary Data 1 [file 42003_2021_2319_MOESM6_ESM.zip › supplementary_data_publication/Fig. 2c.csv.png]

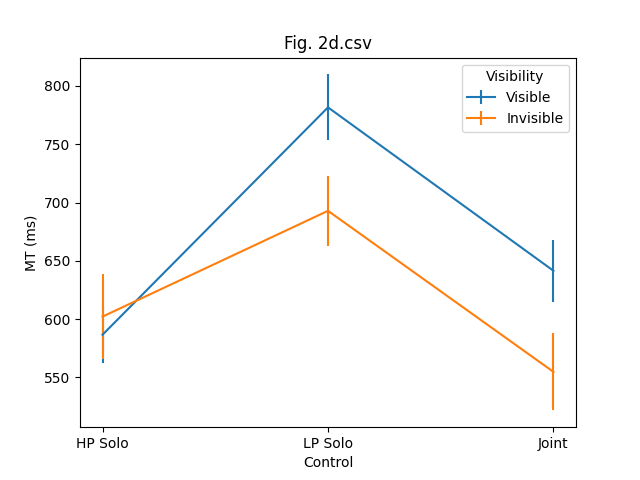

Supplement: Supplementary file 6 — Supplementary Data 1 [file 42003_2021_2319_MOESM6_ESM.zip › supplementary_data_publication/Fig. 2d.csv.png]

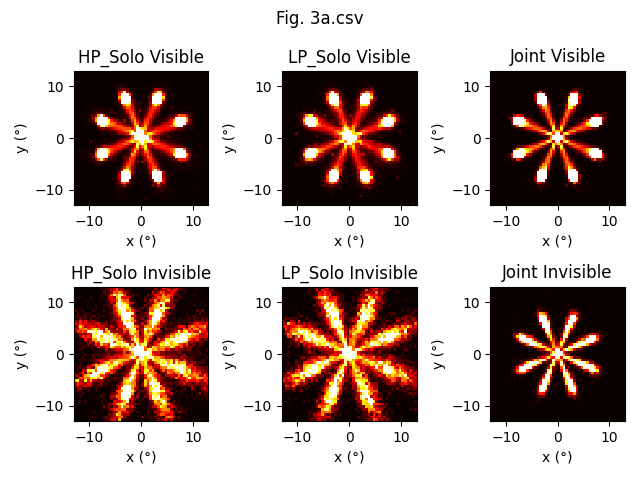

Supplement: Supplementary file 6 — Supplementary Data 1 [file 42003_2021_2319_MOESM6_ESM.zip › supplementary_data_publication/Fig. 3a.csv.png]

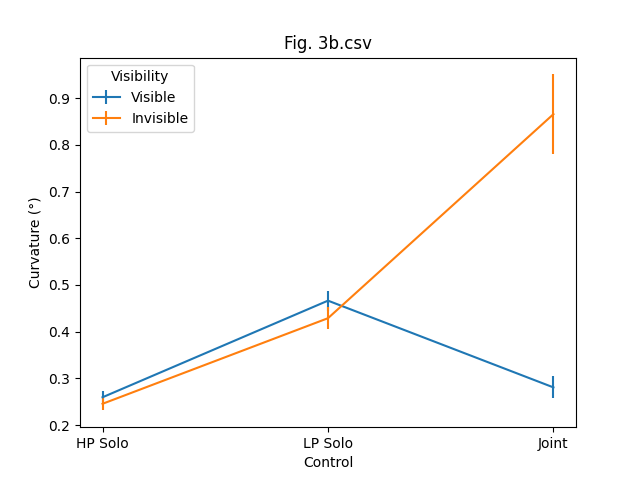

Supplement: Supplementary file 6 — Supplementary Data 1 [file 42003_2021_2319_MOESM6_ESM.zip › supplementary_data_publication/Fig. 3b.csv.png]

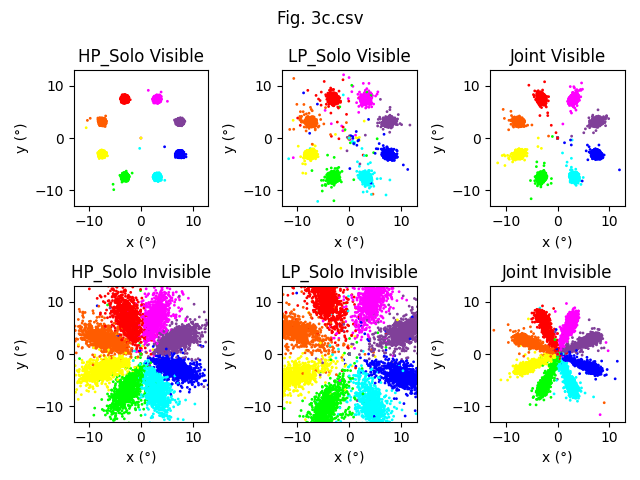

Supplement: Supplementary file 6 — Supplementary Data 1 [file 42003_2021_2319_MOESM6_ESM.zip › supplementary_data_publication/Fig. 3c.csv.png]

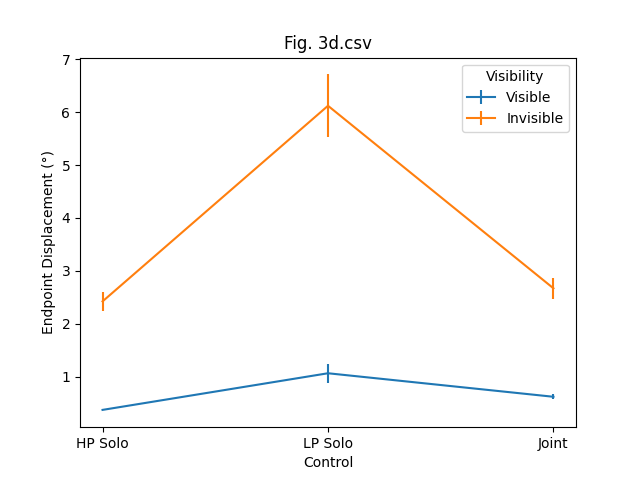

Supplement: Supplementary file 6 — Supplementary Data 1 [file 42003_2021_2319_MOESM6_ESM.zip › supplementary_data_publication/Fig. 3d.csv.png]

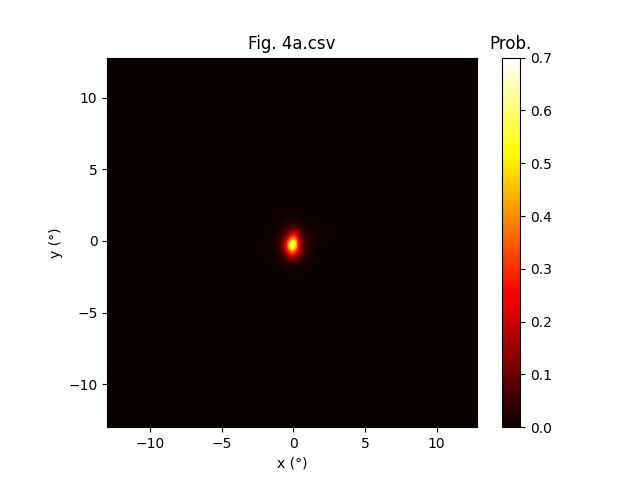

Supplement: Supplementary file 6 — Supplementary Data 1 [file 42003_2021_2319_MOESM6_ESM.zip › supplementary_data_publication/Fig. 4a.csv.png]

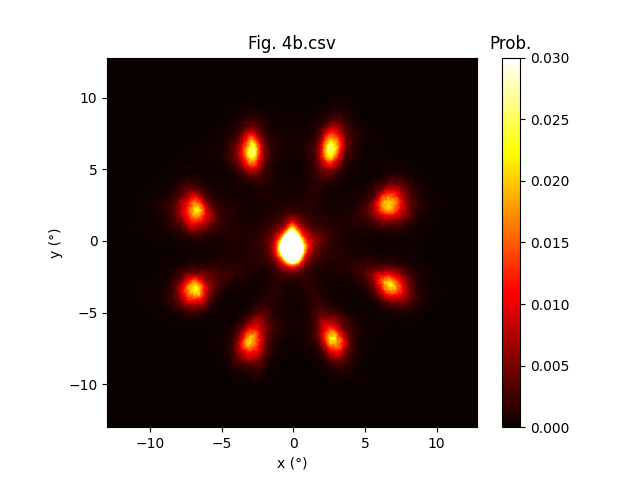

Supplement: Supplementary file 6 — Supplementary Data 1 [file 42003_2021_2319_MOESM6_ESM.zip › supplementary_data_publication/Fig. 4b.csv.png]

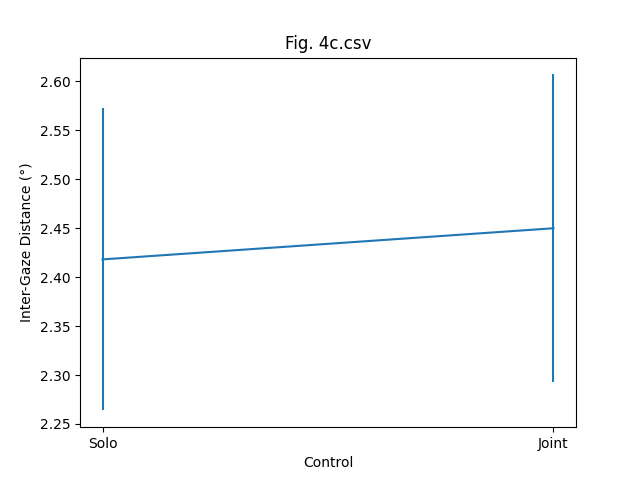

Supplement: Supplementary file 6 — Supplementary Data 1 [file 42003_2021_2319_MOESM6_ESM.zip › supplementary_data_publication/Fig. 4c.csv.png]

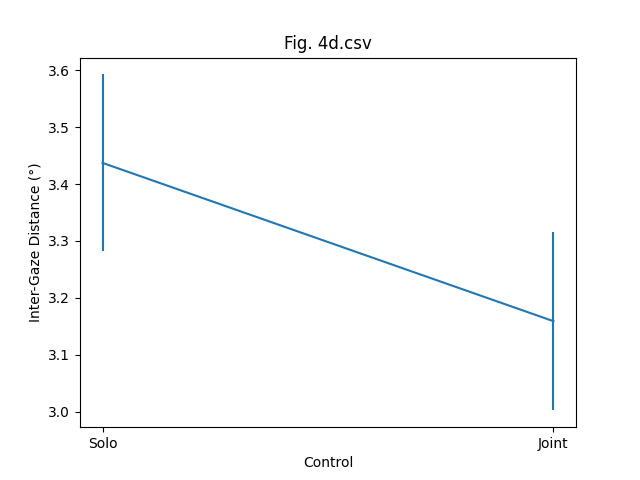

Supplement: Supplementary file 6 — Supplementary Data 1 [file 42003_2021_2319_MOESM6_ESM.zip › supplementary_data_publication/Fig. 4d.csv.png]

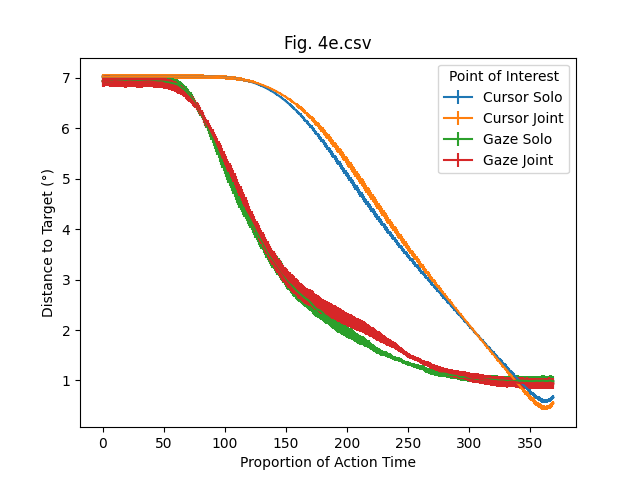

Supplement: Supplementary file 6 — Supplementary Data 1 [file 42003_2021_2319_MOESM6_ESM.zip › supplementary_data_publication/Fig. 4e.csv.png]

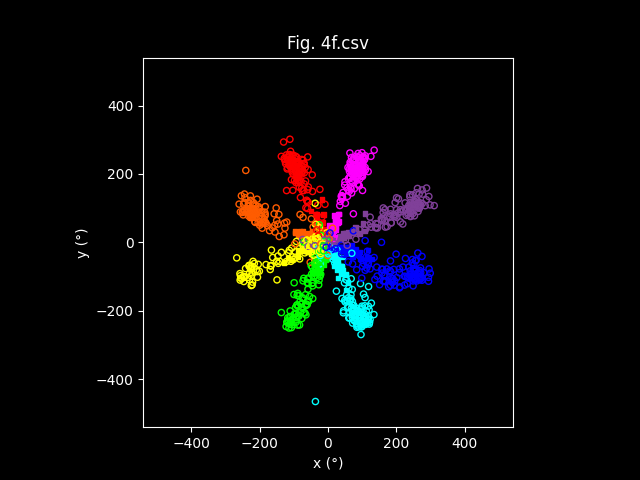

Supplement: Supplementary file 6 — Supplementary Data 1 [file 42003_2021_2319_MOESM6_ESM.zip › supplementary_data_publication/Fig. 4f.csv.png]

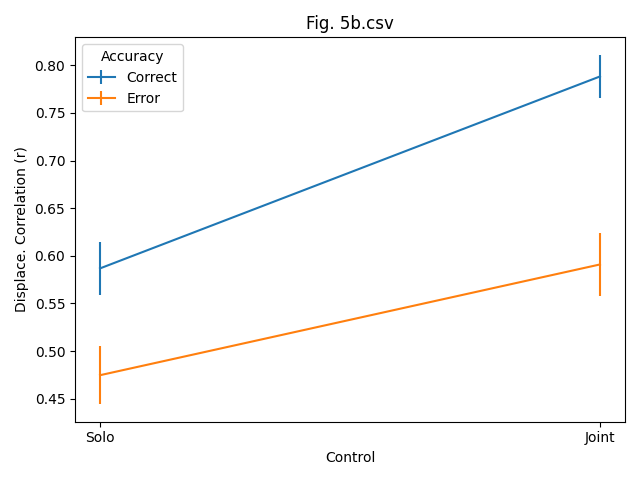

Supplement: Supplementary file 6 — Supplementary Data 1 [file 42003_2021_2319_MOESM6_ESM.zip › supplementary_data_publication/Fig. 5b.csv.png]

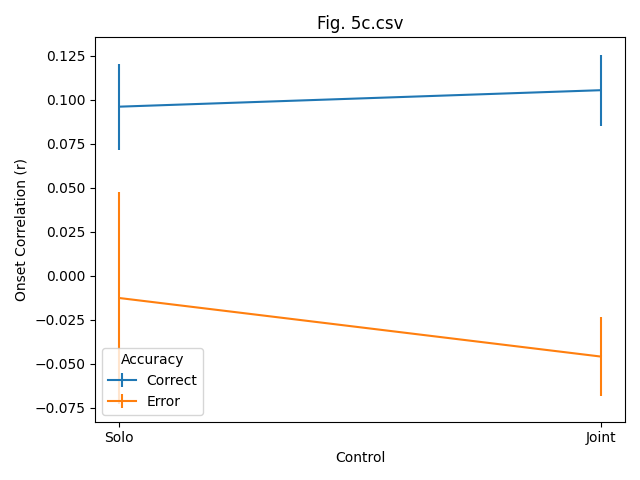

Supplement: Supplementary file 6 — Supplementary Data 1 [file 42003_2021_2319_MOESM6_ESM.zip › supplementary_data_publication/Fig. 5c.csv.png]

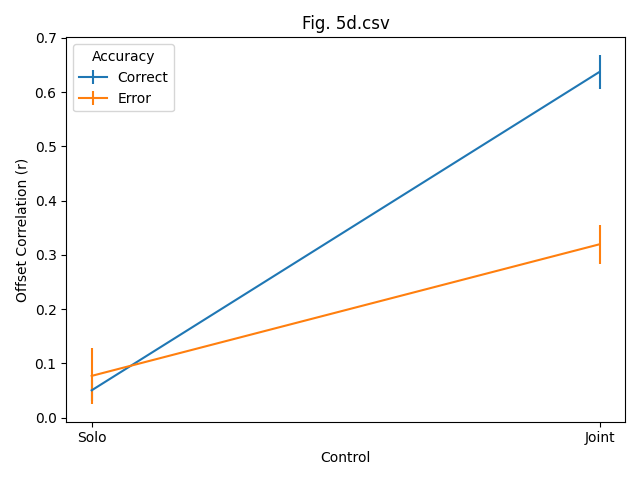

Supplement: Supplementary file 6 — Supplementary Data 1 [file 42003_2021_2319_MOESM6_ESM.zip › supplementary_data_publication/Fig. 5d.csv.png]

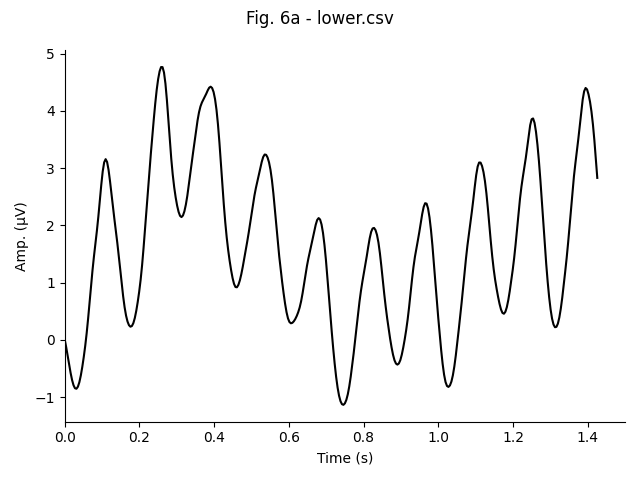

Supplement: Supplementary file 6 — Supplementary Data 1 [file 42003_2021_2319_MOESM6_ESM.zip › supplementary_data_publication/Fig. 6a - lower.csv.png]

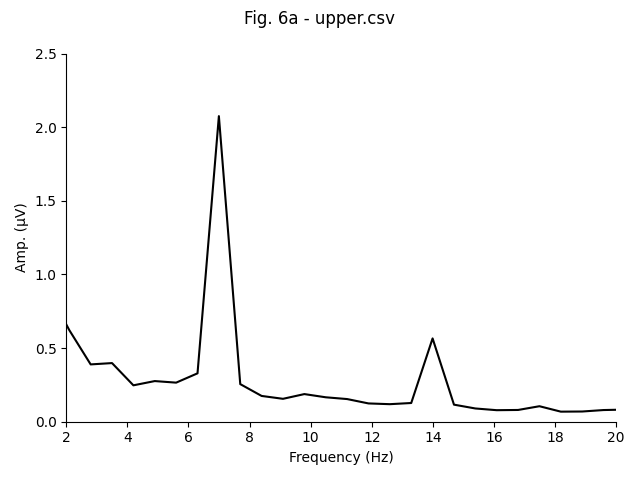

Supplement: Supplementary file 6 — Supplementary Data 1 [file 42003_2021_2319_MOESM6_ESM.zip › supplementary_data_publication/Fig. 6a - upper.csv.png]

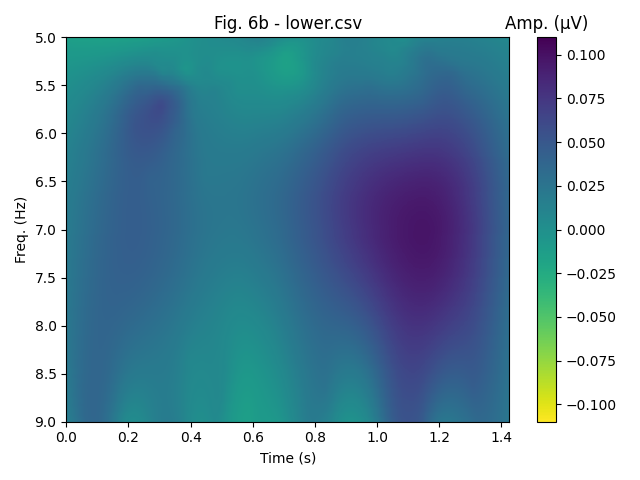

Supplement: Supplementary file 6 — Supplementary Data 1 [file 42003_2021_2319_MOESM6_ESM.zip › supplementary_data_publication/Fig. 6b - lower.csv.png]

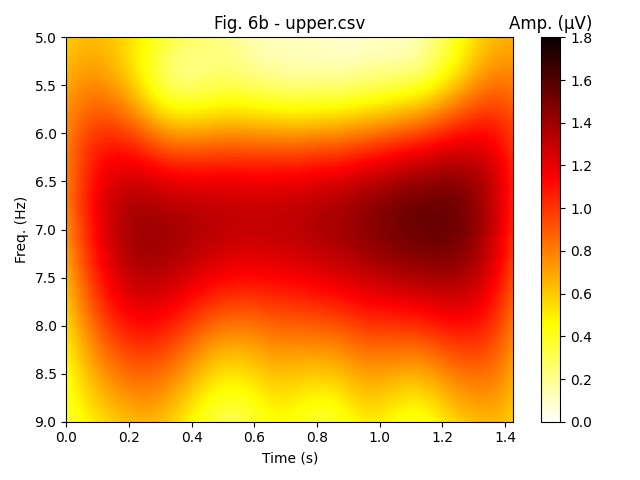

Supplement: Supplementary file 6 — Supplementary Data 1 [file 42003_2021_2319_MOESM6_ESM.zip › supplementary_data_publication/Fig. 6b - upper.csv.png]

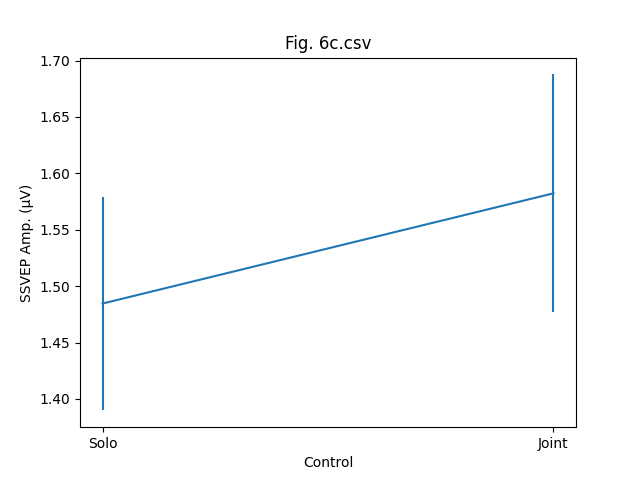

Supplement: Supplementary file 6 — Supplementary Data 1 [file 42003_2021_2319_MOESM6_ESM.zip › supplementary_data_publication/Fig. 6c.csv.png]

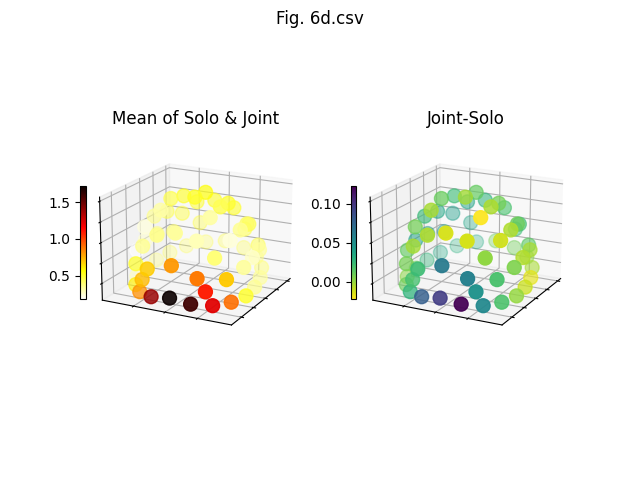

Supplement: Supplementary file 6 — Supplementary Data 1 [file 42003_2021_2319_MOESM6_ESM.zip › supplementary_data_publication/Fig. 6d.csv.png]

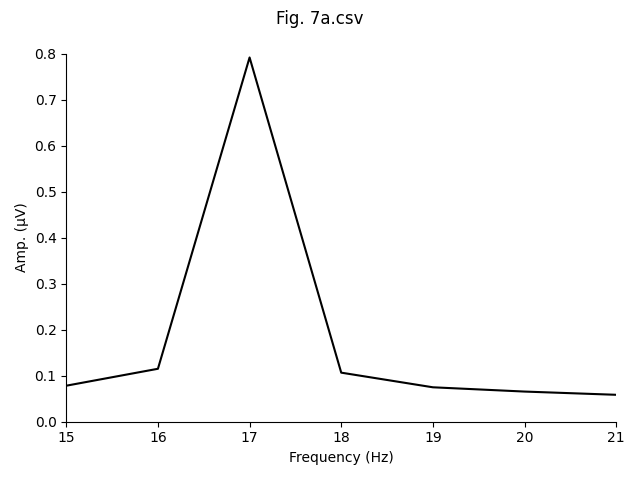

Supplement: Supplementary file 6 — Supplementary Data 1 [file 42003_2021_2319_MOESM6_ESM.zip › supplementary_data_publication/Fig. 7a.csv.png]

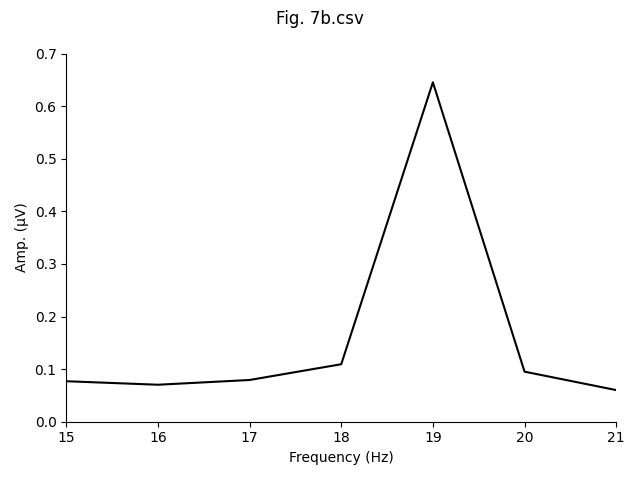

Supplement: Supplementary file 6 — Supplementary Data 1 [file 42003_2021_2319_MOESM6_ESM.zip › supplementary_data_publication/Fig. 7b.csv.png]

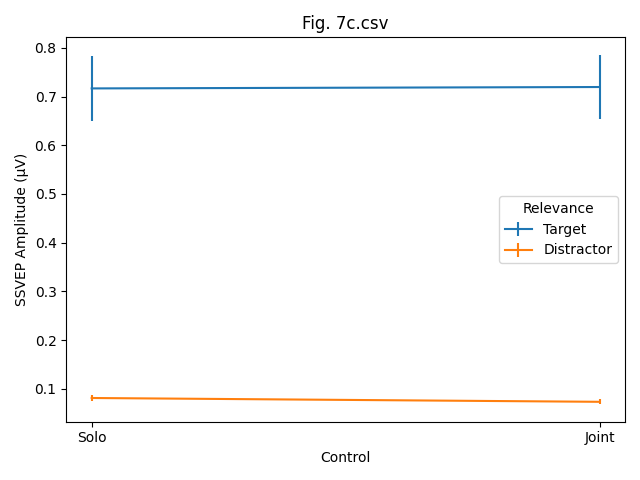

Supplement: Supplementary file 6 — Supplementary Data 1 [file 42003_2021_2319_MOESM6_ESM.zip › supplementary_data_publication/Fig. 7c.csv.png]

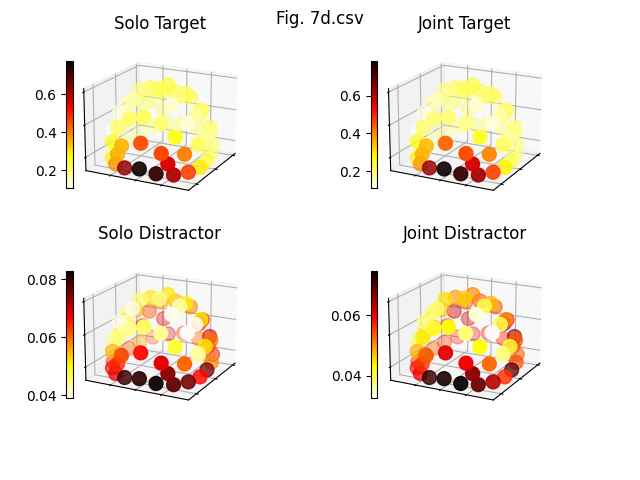

Supplement: Supplementary file 6 — Supplementary Data 1 [file 42003_2021_2319_MOESM6_ESM.zip › supplementary_data_publication/Fig. 7d.csv.png]

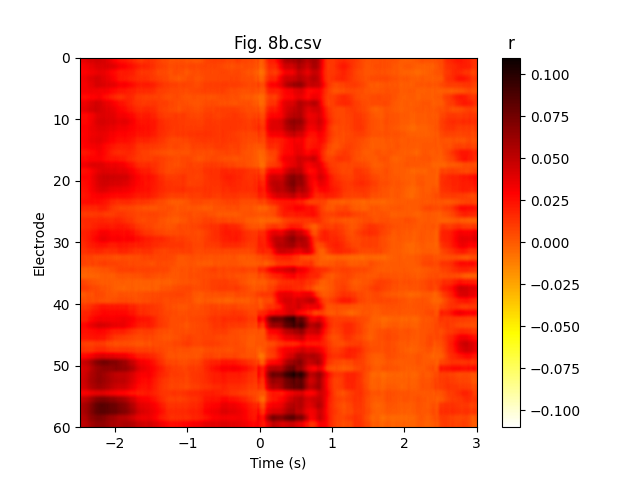

Supplement: Supplementary file 6 — Supplementary Data 1 [file 42003_2021_2319_MOESM6_ESM.zip › supplementary_data_publication/Fig. 8b.csv.png]

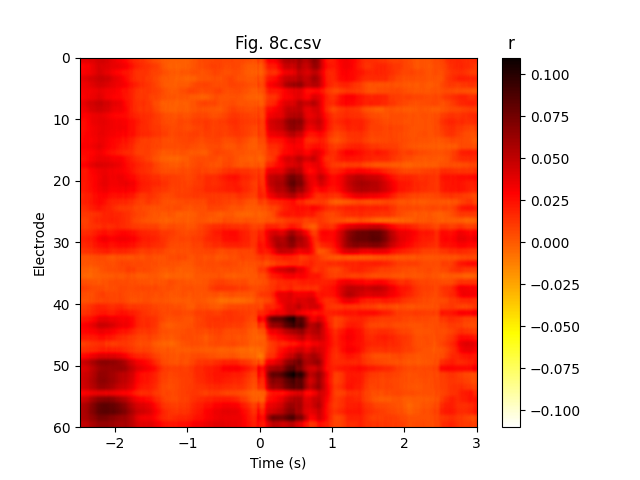

Supplement: Supplementary file 6 — Supplementary Data 1 [file 42003_2021_2319_MOESM6_ESM.zip › supplementary_data_publication/Fig. 8c.csv.png]

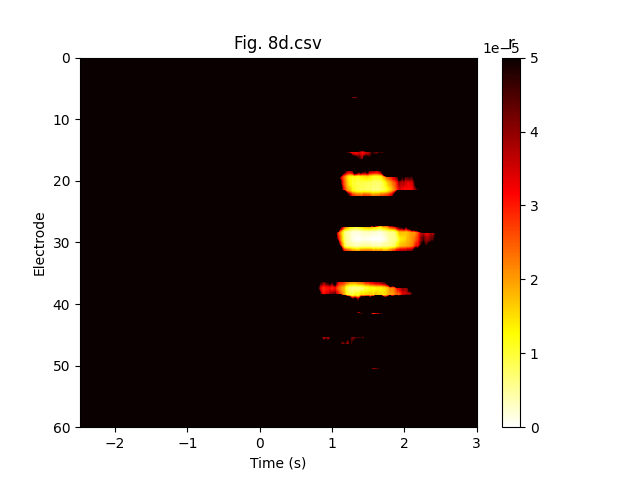

Supplement: Supplementary file 6 — Supplementary Data 1 [file 42003_2021_2319_MOESM6_ESM.zip › supplementary_data_publication/Fig. 8d.csv.png]

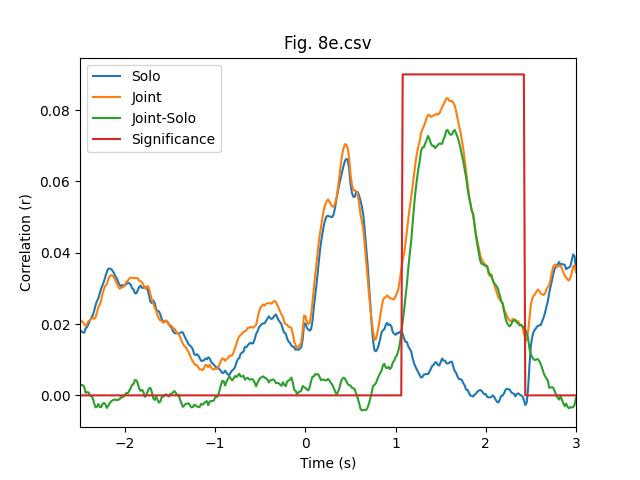

Supplement: Supplementary file 6 — Supplementary Data 1 [file 42003_2021_2319_MOESM6_ESM.zip › supplementary_data_publication/Fig. 8e.csv.png]

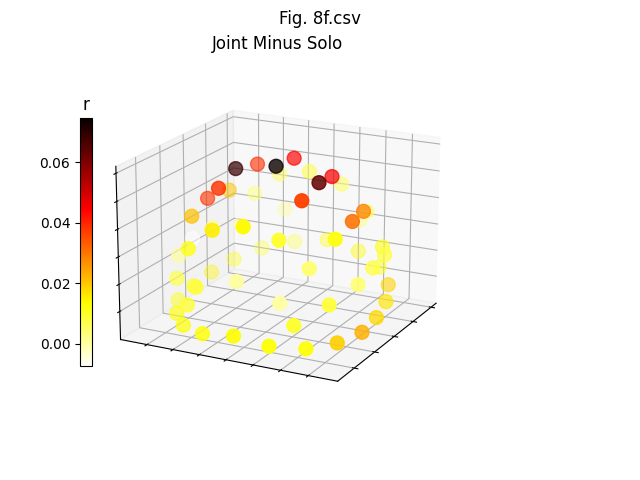

Supplement: Supplementary file 6 — Supplementary Data 1 [file 42003_2021_2319_MOESM6_ESM.zip › supplementary_data_publication/Fig. 8f.csv.png]

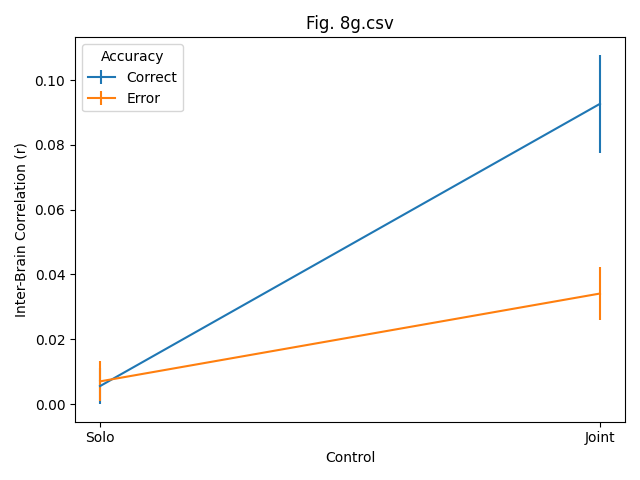

Supplement: Supplementary file 6 — Supplementary Data 1 [file 42003_2021_2319_MOESM6_ESM.zip › supplementary_data_publication/Fig. 8g.csv.png]

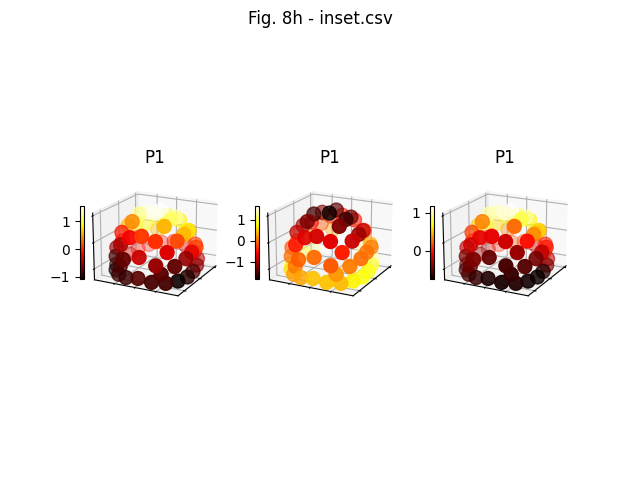

Supplement: Supplementary file 6 — Supplementary Data 1 [file 42003_2021_2319_MOESM6_ESM.zip › supplementary_data_publication/Fig. 8h - inset.csv.png]

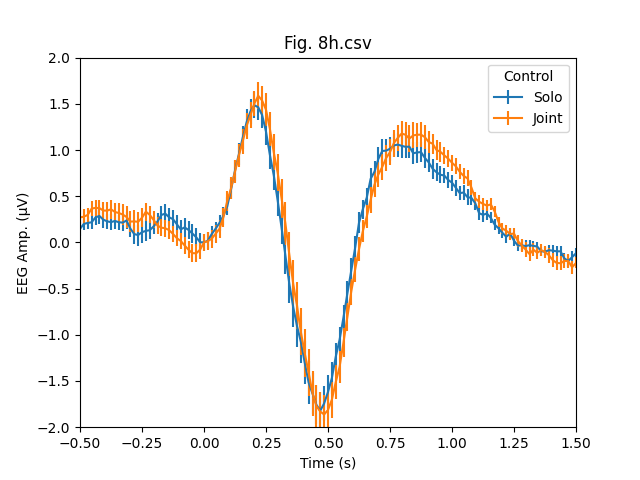

Supplement: Supplementary file 6 — Supplementary Data 1 [file 42003_2021_2319_MOESM6_ESM.zip › supplementary_data_publication/Fig. 8h.csv.png]

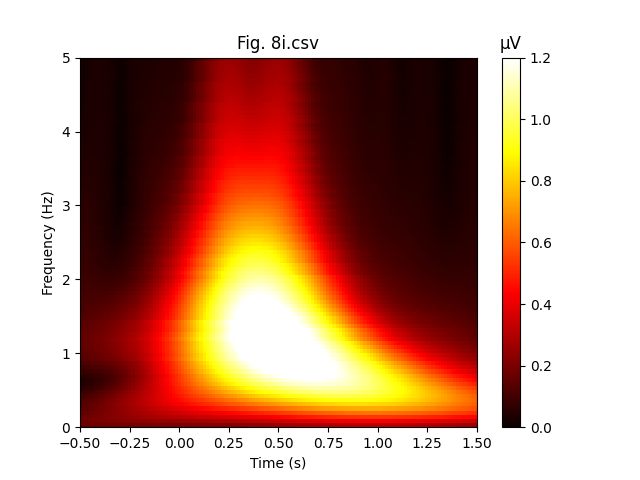

Supplement: Supplementary file 6 — Supplementary Data 1 [file 42003_2021_2319_MOESM6_ESM.zip › supplementary_data_publication/Fig. 8i.csv.png]

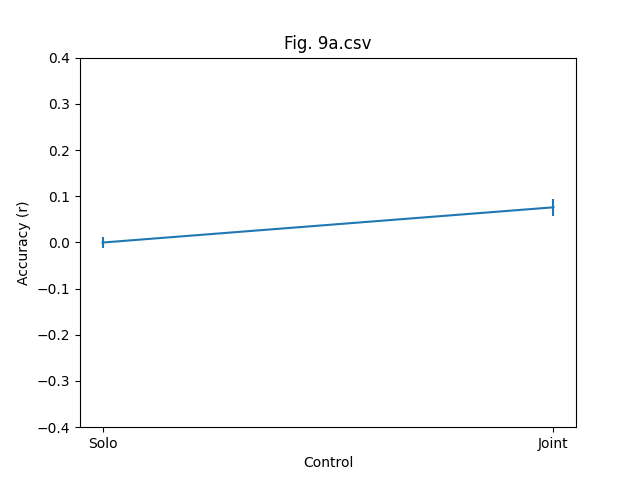

Supplement: Supplementary file 6 — Supplementary Data 1 [file 42003_2021_2319_MOESM6_ESM.zip › supplementary_data_publication/Fig. 9a.csv.png]

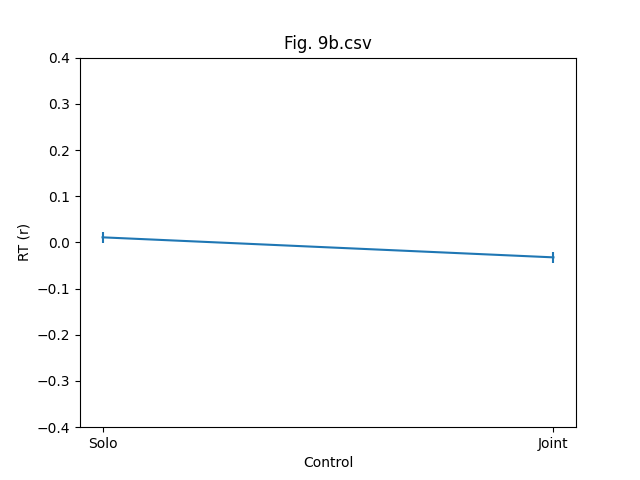

Supplement: Supplementary file 6 — Supplementary Data 1 [file 42003_2021_2319_MOESM6_ESM.zip › supplementary_data_publication/Fig. 9b.csv.png]

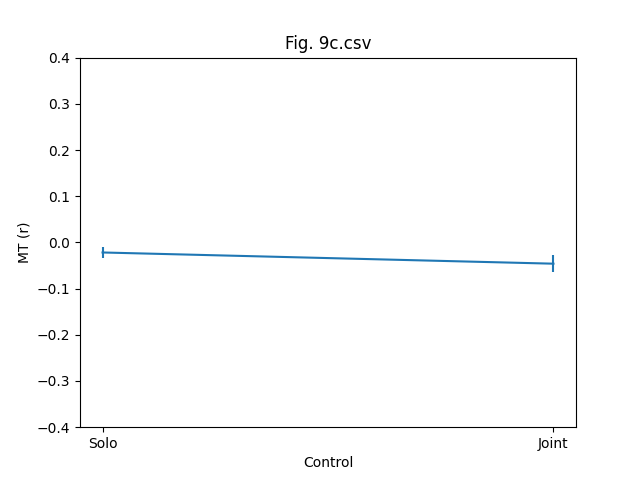

Supplement: Supplementary file 6 — Supplementary Data 1 [file 42003_2021_2319_MOESM6_ESM.zip › supplementary_data_publication/Fig. 9c.csv.png]

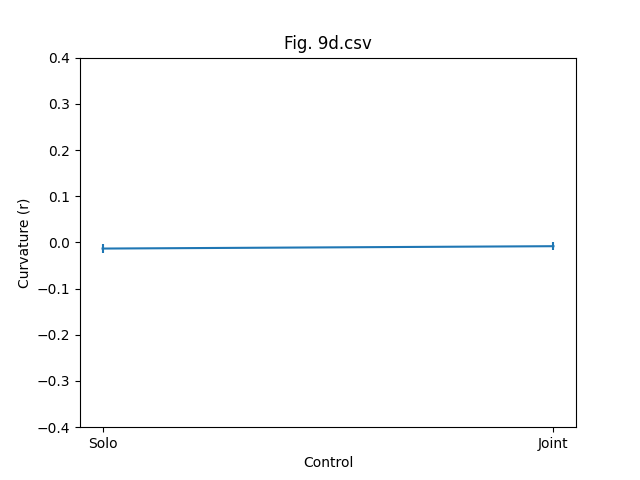

Supplement: Supplementary file 6 — Supplementary Data 1 [file 42003_2021_2319_MOESM6_ESM.zip › supplementary_data_publication/Fig. 9d.csv.png]

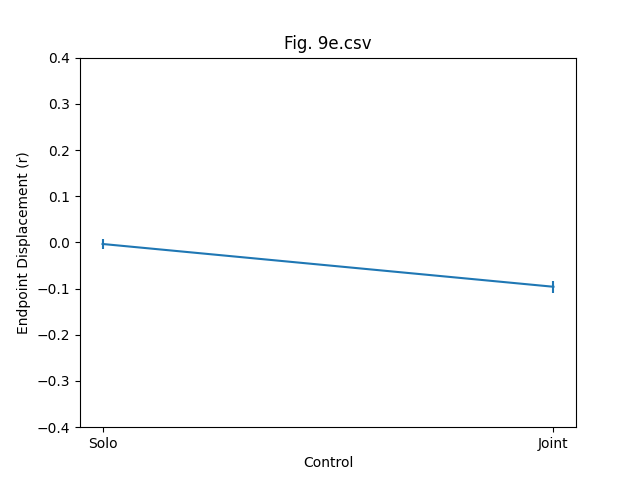

Supplement: Supplementary file 6 — Supplementary Data 1 [file 42003_2021_2319_MOESM6_ESM.zip › supplementary_data_publication/Fig. 9e.csv.png]

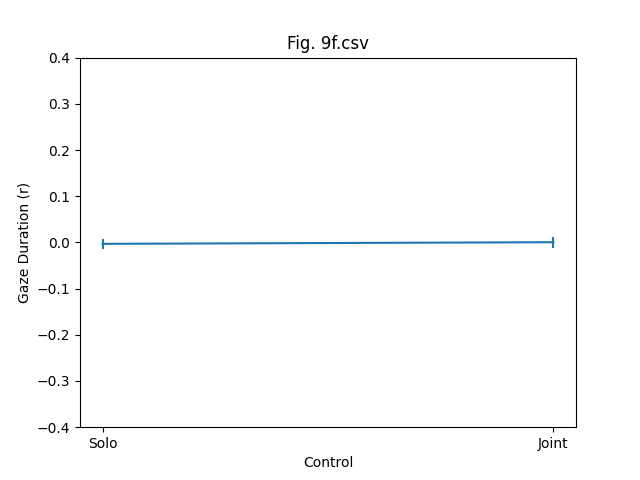

Supplement: Supplementary file 6 — Supplementary Data 1 [file 42003_2021_2319_MOESM6_ESM.zip › supplementary_data_publication/Fig. 9f.csv.png]

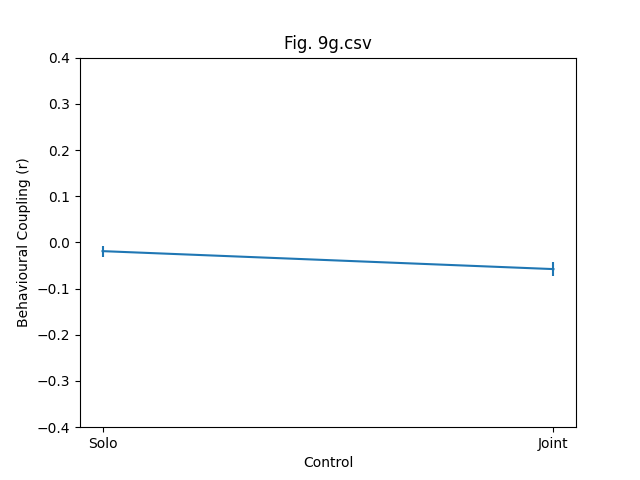

Supplement: Supplementary file 6 — Supplementary Data 1 [file 42003_2021_2319_MOESM6_ESM.zip › supplementary_data_publication/Fig. 9g.csv.png]

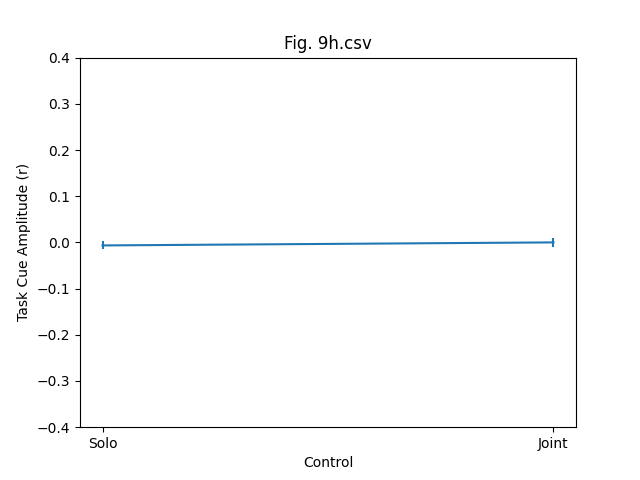

Supplement: Supplementary file 6 — Supplementary Data 1 [file 42003_2021_2319_MOESM6_ESM.zip › supplementary_data_publication/Fig. 9h.csv.png]

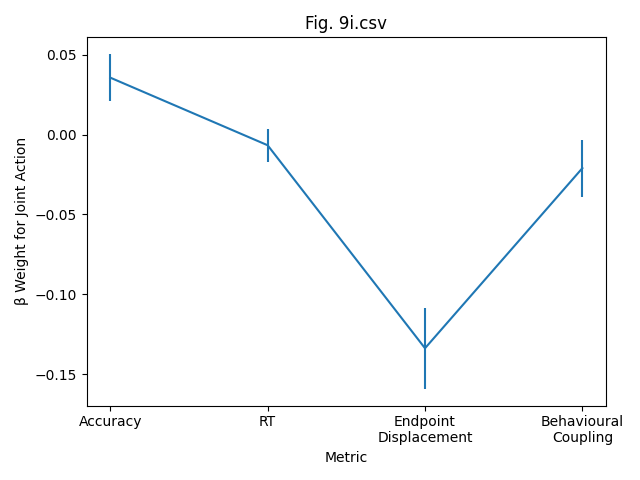

Supplement: Supplementary file 6 — Supplementary Data 1 [file 42003_2021_2319_MOESM6_ESM.zip › supplementary_data_publication/Fig. 9i.csv.png]
