# Supplementary material for: Joint control of visually guided actions involves concordant increases in behavioural and neural coupling
Source: Commun Biol. 2021 Jun 29;4:816. doi: 10.1038/s42003-021-02319-3 (PMC8242020; doi:10.1038/s42003-021-02319-3)
Supplement: Supplementary file 7 — Reporting Summary [file 42003_2021_2319_MOESM7_ESM.pdf]

nature research

Corresponding author(s): Dr David R. Painter

Last updated by author(s): Apr 30, 2021

Reporting Summary

Nature Research wishes to improve the reproducibility of the work that we publish. This form provides structure for consistency and transparency in reporting. For further information on Nature Research policies, see our Editorial Policies and the Editorial Policy Checklist.

Statistics

For all statistical analyses, confirm that the following items are present in the figure legend, table legend, main text, or Methods section.

n/a	Confirmed	
☐	☒	The exact sample size (*n*) for each experimental group/condition, given as a discrete number and unit of measurement
☐	☒	A statement on whether measurements were taken from distinct samples or whether the same sample was measured repeatedly
☐	☒	The statistical test(s) used AND whether they are one- or two-sided<br>*Only common tests should be described solely by name; describe more complex techniques in the Methods section.*
☐	☒	A description of all covariates tested
☐	☒	A description of any assumptions or corrections, such as tests of normality and adjustment for multiple comparisons
☐	☒	A full description of the statistical parameters including central tendency (e.g. means) or other basic estimates (e.g. regression coefficient) AND variation (e.g. standard deviation) or associated estimates of uncertainty (e.g. confidence intervals)
☐	☒	For null hypothesis testing, the test statistic (e.g. *F*, *t*, *r*) with confidence intervals, effect sizes, degrees of freedom and *P* value noted<br>*Give P values as exact values whenever suitable.*
☒	☐	For Bayesian analysis, information on the choice of priors and Markov chain Monte Carlo settings
☒	☐	For hierarchical and complex designs, identification of the appropriate level for tests and full reporting of outcomes
☐	☒	Estimates of effect sizes (e.g. Cohen's *d*, Pearson's *r*), indicating how they were calculated

Our web collection on statistics for biologists contains articles on many of the points above.

Software and code

Policy information about availability of computer code

Data collection	Stimuli were presented at 144 Hz on two ASUS VG248QE LCD monitors synchronised in Eyefinity display mode (combined resolution 3840×1080). Stimuli were presented on a black background (RGB: 0, 0, 0) at a viewing distance of 57 cm using the Cogent 2000 Toolbox (http://www.vislab.ucl.ac.uk/cogent_2000.php) running in MATLAB R2014a (32-bit; Mathworks, Natick, MA) under Windows 7 (64-bit) on a Dell Precision T1700 PC. Data was acquired and exported using ANT Neuro software.
Data analysis	Given that participants were free to move their gaze during the experiment, eye movement artefacts were topographically interpolated with 150 µV horizontal and 250 µV vertical eye action thresholds (BESA v.6.3, MEGIS Software GmbH, Munich, Germany). Analyses were performed using custom-written methods for MATLAB R2015a (64-bit), R (version 4.0.2), Python 3.8.5 (64-bit), and IBM SPSS Statistics Version 21. Raincloud plots were produced using R's ggplot2 library. All other plots were produced using ggplot2 or MATLAB.

For manuscripts utilizing custom algorithms or software that are central to the research but not yet described in published literature, software must be made available to editors and reviewers. We strongly encourage code deposition in a community repository (e.g. GitHub). See the Nature Research guidelines for submitting code & software for further information.

Data

Policy information about availability of data

All manuscripts must include a data availability statement. This statement should provide the following information, where applicable:

- Accession codes, unique identifiers, or web links for publicly available datasets
- A list of figures that have associated raw data
- A description of any restrictions on data availability

Provide your data availability statement here.

Field-specific reporting

Please select the one below that is the best fit for your research. If you are not sure, read the appropriate sections before making your selection.

☐ Life sciences ☒ Behavioural & social sciences ☐ Ecological, evolutionary & environmental sciences

For a reference copy of the document with all sections, see nature.com/documents/nr-reporting-summary-flat.pdf

Behavioural & social sciences study design

All studies must disclose on these points even when the disclosure is negative.

Study description	The study is a quantitative behavioural/neuroimaging experiment.
Research sample	Twenty pairs of participants (total N = 40) volunteered for the study and were recruited from The University of Queensland Psychology Research Participation Scheme. Participants were 18–34 years old (M = 22.33 years, SD = 2.98; 20 males, two left-handed) and reported no personal or familial history of photosensitive epilepsy or seizures, and therefore could safely view the flickering visual displays. Four pairs were both males and four pairs were both females. Participants were paid for three hours at a rate of $10/hour. Pay was not contingent on performance.
Sampling strategy	Participants were pseudo-randomly paired based on their timeslot for participation with the requirement that pairs not be acquaintances. The sample size (N = 20 pairs) was based on previous frequency-tagging investigations of attentional processes.
Data collection	Stimuli were presented at 144 Hz on two ASUS VG248QE LCD monitors synchronised in Eyefinity display mode (combined resolution 3840×1080). Stimuli were presented on a black background (RGB: 0, 0, 0) at a viewing distance of 57 cm using the Cogent 2000 Toolbox (http://www.vislab.ucl.ac.uk/cogent_2000.php) running in MATLAB R2014a (32-bit; Mathworks, Natick, MA) under Windows 7 (64-bit) on a Dell Precision T1700 PC. EEG was sampled at 2000 Hz from 61 scalp channels using a 64-channel amplifier and Ag/AgCl electrodes positioned according to the 10-10 system within a WaveGuard cap (ANT Neuro, Germany). Gaze position was sampled at 120 Hz using an iView Red-m infrared eye tracker (SensoMotoric Instruments, Germany).<br>During testing, no one was present besides the two participants and one to three researchers (required for the dual-EEG setup phase). The researchers were not blind to the experimental conditions and study hypotheses during data collection. It is unlikely that this affected the key results, which were based on correlations between behavioural and neural measures.
Timing	Data collection started on June 7 2016 and ended on August 5 2016.
Data exclusions	Four participants from three participant pairs were excluded for missing or inaccurate eye tracking data. These participants were included in all other analyses. Note that uncorrected acuity was not a requisite for participation in the study, and correction can produce inaccuracies in eye tracking. Such exclusions are commonplace in eye tracking research, and do not affect the main conclusions of the study, which are based on EEG and behaviour.
Non-participation	Zero participants dropped out/declined participation.
Randomization	On randomly interleaved trials, an action control cue changed colour (to green or red), informing participants with 100% validity that they were about to perform a solo or joint action. The action control cue contingency was made explicit to participants, and cue colours were counterbalanced across participant pairs.

Reporting for specific materials, systems and methods

We require information from authors about some types of materials, experimental systems and methods used in many studies. Here, indicate whether each material, system or method listed is relevant to your study. If you are not sure if a list item applies to your research, read the appropriate section before selecting a response.

Materials & experimental systems

n/a	Involved in the study	
☒	☐	Antibodies
☒	☐	Eukaryotic cell lines
☒	☐	Palaeontology and archaeology
☒	☐	Animals and other organisms
☐	☒	Human research participants
☒	☐	Clinical data
☒	☐	Dual use research of concern

Methods

n/a	Involved in the study	
☒	☐	ChIP-seq
☒	☐	Flow cytometry
☒	☐	MRI-based neuroimaging

Human research participants

Policy information about studies involving human research participants

Population characteristics	Participants were 18–34 years old (M = 22.33 years, SD = 2.98; 20 males, two left-handed) and reported no personal or familial history of photosensitive epilepsy or seizures, and therefore could safely view the flickering visual displays. Four pairs were both males and four pairs were both females.
Recruitment	Twenty pairs of participants (total N = 40) volunteered for the study and were recruited from The University of Queensland Psychology Research Participation Scheme. Self-selection and other biases are not likely to have impacted the results.
Ethics oversight	The University of Queensland Human Research Ethics Committee approved the study protocol, and all participants provided informed written consent.

Note that full information on the approval of the study protocol must also be provided in the manuscript.
